# Draft Genome Sequence of *Kerstersia gyiorum* CG1, Isolated from a Leg Ulcer

**DOI:** 10.1128/genomeA.01036-15

**Published:** 2015-09-10

**Authors:** Alexander L. Greninger, Varvara Kozyreva, Chau-Linda Truong, Rose Longoria, Vishnu Chaturvedi

**Affiliations:** Microbial Diseases Laboratory, California Department of Public Health, Richmond, California, USA

## Abstract

We report the first draft genome sequence of *Kerstersia gyiorum* from a leg ulcer of a patient with diabetes and osteomyelitis. The 3.94-Mb genome assembly included 3,428 annotated coding sequences with an *N*_50_ of 223,310 bp and a plasmid encoding a type IV secretion system gene and two antitoxin genes.

## GENOME ANNOUNCEMENT

*Kerstersia gyiorum* is a Gram-negative bacterium of the order *Burkholderiales* that is occasionally isolated from clinical samples. The *Kerstersia* genus was described in 2003 based on a polyphasic taxonomic study of *Alcaligenes faecalis*–like organisms that formed a unique clade (1). *Kerstersia* members are biochemically very similar to the *Alcaligenaceae* and can only be speciated by sequence, fatty acid composition, and whole-cell protein lysates. Isolation of *K. gyiorum* has been reported from a total of five cases of chronic otitis media and eight lower extremity wound infections (1–4). Recently, the first cases of *K. gyiorum* bacteremia and bronchoalveolar lavage fluid isolations were reported (5, 6).

Because of the lack of a genomic sequence from any members of the genus *Kerstersia*, we sequenced the first draft genome of *K. gyiorum* strain CG1. This strain originated from a leg ulcer specimen from a 57-year-old man with a history of diabetes and osteomyelitis. The isolate was identified as a *K. gyiorum* in our laboratory, based on partial 16S rRNA sequencing, in addition to MALDI-TOF MS analysis.

DNA from *K. gyiorum* was extracted using the Promega Genomic DNA Wizard kit, and paired-end libraries were constructed using the Nextera XT kit. Sequences were adapted and quality (Q30) trimmed using Cutadapt, *de novo* assembled using SPAdes version 3.5, and annotated via Prokka version 1.1 (7–10). A total of 5,228,748 paired-end reads with an average length of 208 nucleotides were recovered after trimming. *De novo* assembly yielded 32 contigs covering a total of 3,943,213 bp with an *N*_50_ of 223,310 bp, an average coverage of 260×, and a total of 3,428 annotated coding sequences. The assembled 16S sequence aligned 100% to *K. gyiorum* S7. Coverage of the rRNA locus suggests that there are likely five copies of rRNA dispersed throughout the genome.

Strain CG1 possesses a 9,094-nucleotide plasmid with 9× greater coverage than the genome and homology to *Xanthomonas* and *Achromobacter* spp. plasmids. The plasmid encodes a virB6 type IV secretion system with a putative adenylate kinase–like protein opposed by a VbhA-like antitoxin coding sequence. The plasmid also encodes a second antitoxin sequence in a pemI-like gene. The remaining sequence of the plasmid is devoted to repA (70% amino acid to *Paracoccus* sp. TRP), ParA, and DNA-invertase hin, along with 5 hypothetical proteins. Antibiotic resistance genes encoded on the chromosome included an aminoglycoside adenylyltransferase (69% amino acid to *Mannheimia haemolytica* MhSwine 2000) and a chloramphenicol acetyltransferase (80% amino acid to *Morganella morganii* MM1).

### Nucleotide sequence accession number. 

This whole-genome shotgun project has been deposited at DDBJ/EMBL/GenBank under the accession number LBNE00000000.
